# Self-Administered Virtual Reality for Postsurgical Pain Management: A Qualitative Study of Hospital Patients’ Reported Experiences

**DOI:** 10.3390/jcm12216805

**Published:** 2023-10-27

**Authors:** Elisabeth J. Lier, Merlijn L. M. Smits, Marjan de Vries, Harry van Goor

**Affiliations:** 1 Department of Surgery, Radboud University Medical Center, 6500 HB Nijmegen, The Netherlands; janienkelier@gmail.com (E.J.L.); m.l.m.smits@saxion.nl (M.L.M.S.); marjan.devries@radboudumc.nl (M.d.V.); 2 Department of Anaesthesiology, Pain and Palliative Medicine, Radboud University Medical Center, 6500 HB Nijmegen, The Netherlands; 3 Department of Industrial Design, Saxion University of Applied Sciences, 7500 KB Enschede, The Netherlands

**Keywords:** Virtual Reality, multimodal pain management, postsurgical pain management

## Abstract

Virtual Reality (VR) has been shown to effectively reduce pain in patients with various pain conditions. However, questions arise on the use of VR in multimodal postsurgical pain management. Optimizing VR for pain management requires an understanding of intervention- and context-specific factors, based on patients’ needs and expectations after major surgery. This substudy is part of a randomized controlled trial investigating the effects of three VR interventions as an add-on, self-administered treatment for postsurgical pain. Semi-structured interviews were conducted to evaluate VR effects, software, hardware, prescriptions, and factors affecting the implementation of VR. Experiences across interventions were compared to identify relevant factors for successful implementation. Patients benefitted from self-administered VR in postsurgical pain management in various aspects and without serious drawbacks. Participants preferred an intuitive, 3D, 360-degree VR device with a large choice of applications matching their interests. The preferred frequency and duration of VR use was 2–3 sessions a day for 10–15 min each. Adjusting the VR use to individuals’ needs and contexts was reported to be key for successful implementation, with attention paid to improving the awareness of VR as a non-pharmacological means of promoting postsurgical recovery among patients and healthcare professionals.

## 1. Introduction

The application of Virtual Reality (VR) as a means to manage pain was initially applied in procedural pain during burn wound care, and it has expanded considerably in recent years. VR has been studied extensively in various pain conditions and has proven effective in relieving acute, chronic, and procedural pain [1,2,3,4]. VR provides pain relief through various mechanisms; however, VR analgesia is mainly attributed to distracting attention from pain. Experiencing VR and pain perception both require attention, but due to restricted cognitive attentional capacity, this competition for cognitive resources results in less attention being available for pain processing and, consequently, an altered pain experience [5,6]. Despite its reported effectiveness, VR is not routinely used in pain management. Many questions exist on the use of VR, specifically in multimodal postsurgical pain management. Little is known about patients’ experiences, recommendations on VR for pain management after major surgery, and which intervention- and context-specific factors are relevant for VR use and implementation.

For example, with regard to intervention-specific factors, healthcare institutions should decide which VR headsets and applications to offer to patients. Previous research has found that some hardware features, such as a high-degree field-of-view and high levels of interaction, are associated with increased pain reduction, but the impact of such features on patients’ experiences and ease of use is unknown [7,8,9,10]. Also, it is unclear how the ideal software catalogue could be compiled, and what the preferred number and topics for applications would be. Many VR studies have offered a limited catalogue, as in, a small number of applications or a specific number of topics limited to nature videos or a racing game. It is unclear which VR software content works best from the patient’s perspective and whether preferences for content depend on specific individual factors. In addition, most studies have prescribed VR for a fixed period of time during the day, but limited insight is available into the most appropriate VR prescription for VR dose and frequency in postsurgical, hospitalized patients. Since VR has mostly been applied as an intervention supervised by a researcher, more insight is needed into the use of VR as a self-administered intervention that can be applied whenever the patient requires (unscheduled) pain therapy. By determining the optimal VR technology and prescription, this study could contribute to a VR therapy that is more in concordance with patient’s desires and expectations, as well as being more effective [11,12,13].

For context-specific factors, little is known about which factors should be considered when implementing VR in normal postoperative care. For example, it is unclear which factors hinder the use of VR in current care routines for clinical patients and whether VR requires specific support from healthcare personnel and technical assistants.

In this paper, we aim to optimize VR according to the needs and expectations of clinical patients by evaluating which intervention- and context-specific factors are relevant for successful implementation. We investigate patients’ experiences of self-administered VR in the first postoperative days and study their preferences for VR hardware, software, and prescriptions. These preferences result in recommendations on the optimal use and implementation of VR.

## 2. Materials and Methods

This study is a substudy of the VIRTUAL trial, a randomized controlled trial on the effect of VR on postsurgical pain and recovery (registered at ClinicalTrials.gov NCT03933124). This multicenter RCT was conducted at the Radboud University Medical Center (university hospital) and the Sint Maartenskliniek (tertiary referral hospital for orthopedic surgery), in Nijmegen, the Netherlands, from May 2019 to May 2021. Participating departments were gastrointestinal, trauma, cardiothoracic, orthopedic, urological, gynecological, and plastic surgery.

A comprehensive methodological description of the study and results on the effectiveness of VR on clinical outcomes have been reported separately. In short, postsurgical adult patients who underwent surgery with an estimated length of stay of at least four days and reported a pain score of 4 or higher were included on the first day after surgery. Patients were excluded if they suffered from delirium or an acute confusional state; a history of dementia, seizure, or epilepsy; severe hearing/visual impairment; isolation precautions or head wounds; or if they were (re)admitted to the intensive care unit; if they were included in other clinical trials investigating pain therapies; or if they were not able to understand or comply with the study protocol (for example, patients not speaking Dutch). Patients were randomized to one of three different VR groups or a control group, with a total sample size of 100 participants. All participants provided written informed consent. 

### 2.1. Participants

Patients randomized to one of the VR groups were asked separately to participate in this interview study. Patient inclusion for this substudy started after 20 participants were enrolled in the trial to avoid unforeseen issues affecting the conduct and outcomes of this substudy, such as technical aspects concerning the VR devices or patient support issues that were not covered during pilot testing prior to initiation of the trial. The sample size was determined by data saturation, defined as the moment when additional interviews would not result in new information or themes. We aimed to include at least 15 interviews, including 5 interviews per intervention subgroup, before conducting analyses to check for data saturation.

### 2.2. Interventions

In this study, VR was defined as any intervention provided by a head-mounted display (HMD), covering (part of) the patient’s view of the hospital environment. Participants randomized for VR were stratified into one of three subgroups with different hardware and software specifications (Table 1). For hardware, this included differences between two-dimensional and three-dimensional fields of view, as well as non-immersive and immersive devices. Software applications varied between non-interactive nature videos and highly interactive VR games, as specified in Table 1.

All patients received standard care and pain management during the postsurgical course. Standardized stepwise analgesic treatment consisted of paracetamol, non-steroidal anti-inflammatory drugs (NSAIDs) if not contraindicated, and opioids, both short acting and long acting (oxycodone, morphine, and piritramide). Patients who underwent visceral surgery or orthopedic surgery routinely received patient-controlled intravenous analgesia (PCIA) and/or epidural analgesia with morphine derivates, unless contraindicated. VR was used as an add-on self-administered tool in addition to standard care and pain management. On the first day after surgery, participants received a supervised training session with the HMD for approximately 15 min. Participants were advised to use VR unsupervised at least 3 times a day for approximately 10 min per session, from postoperative day 2 to 4. However, they were allowed to taper the VR use to their needs, choosing the time and duration of the VR applications without supervision from clinicians or researchers. To this end, the HMD was placed near the bed around the clock. Participants received a manual for the device, including a section on common technical issues. A support service provided by members of the research team was available at the surgical ward to assist patients when needed.

### 2.3. Data Collection and Analysis

Patients who agreed to participate were interviewed face to face in a semi-structured interview just before discharge from the hospital. Interviews were conducted in private rooms or private spaces. The interview, conducted by a member of the research team, focused on users’ experiences and recommendations concerning the following predefined topics: VR effects, software and hardware, and patients’ recommendations on VR prescriptions and the implementation of VR (Supplementary Materials S1). All interviews were audio-recorded and transcribed verbatim directly after the interviews. To minimize bias, two independent researchers with a background in medicine (EJL) and industrial design engineering (MLMS) performed a thematic analysis to systematically analyze the interview data [14]. During the initial and open coding process, the researchers separately analyzed the data from the first 15 interviews and identified themes. Interview data were grouped into similar themes using Microsoft Word. After identifying the initial themes, the two researchers compared and discussed themes and brought them together into one consistent set of themes. Discrepancies were discussed until a consensus was reached. Via selective coding, all interview data were consequently grouped into the defined set of themes. Per main topic and theme, the three VR types were compared. Standards for reporting qualitative research were followed to ensure study trustworthiness. 

**Figure 1:**
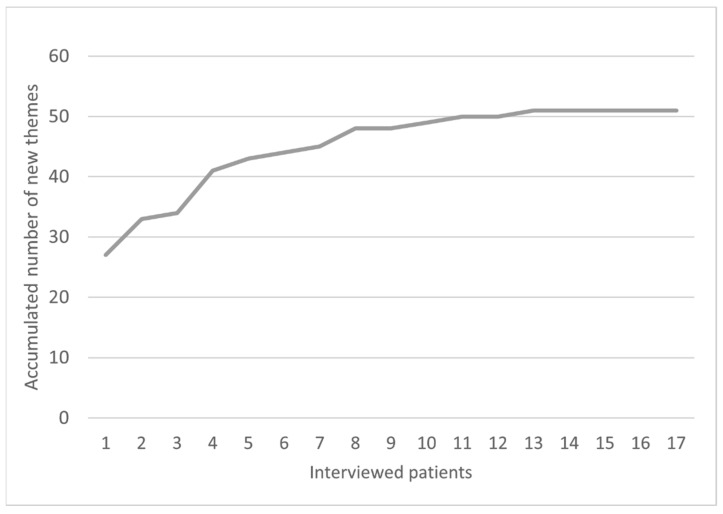
Data saturation of the interviews. X-axis represents number of patients interviewed; Y-axis represents the accumulated number of new themes mentioned by patients.

To determine data saturation, defined as the moment when additional interviews would not result in new information or themes, all themes were listed in Microsoft Excel, and all codes belonging to the themes were grouped. Per interview, we listed how many novel themes were introduced compared to the previous interview. Data saturation was assessed using histograms, in which all new factors were shown for each interview. When no new topics or themes were identified, as determined by the two researchers during the thematic analysis, the histograms indicated a horizontal line. After 15 interviews, data saturation was reached (see Figure 1). However, participants were all included in one participating hospital. Therefore, we held additional interviews with participants from the second hospital to check whether a different hospital setting would result in novel themes. No new themes were identified after conducting two additional interviews. Consequently, no further interviews were held.

## 3. Results

### 3.1. Demographics of Interviewed Patients

A total of 27 patients were asked to participate in the interviews, and 17 patients agreed (Table 2). Four patients could not be interviewed since they felt too ill (due to fever, nausea, and fatigue), three patients were discharged before they could be interviewed, and another three patients were not willing to participate. No differences in baseline characteristics were observed between the interviewed participants and the patients not able to or not willing to participate. The interviewed participants included nine men (52.9%) and eight women, with a mean age of 57.9 years (Table 2). The mean number of VR sessions from postoperative day 2 to 4 was 6.5 (SD 3.2), and the total time in VR was 95 min (median, range: 10–315). The interviews had a median duration of 16 min (range: 7–20 min).

All participants were generally positive about the use of VR. Seven participants reported their experiences to be beyond expectations. All participants would recommend VR to other patients. All but one participant would use VR again in the future. Three participants considered buying a VR device themselves, and one had already bought a headset online during their hospital stay for use at home after discharge.

The total number of themes and subthemes identified was 51 (Figure 1, Supplementary Materials S2). These were divided into five topics, including VR effects, VR hardware, VR software, VR prescriptions, and implementation.

### 3.2. VR Effects

Various positive effects of VR were experienced by the participants, including pain reduction, distraction, relaxation, privacy, and immersion. In total, eight participants in three intervention groups experienced less pain due to VR. As participant V030 (62 yr, female, VR intervention group I) described: “*VR has done me a lot of good, really. […] When I was in pain, I could focus on something else*”**. Thirteen participants indicated that VR provided a distraction from pain. This was mostly noted by participants in VR intervention III. Although three participants mentioned the possibility of medication withdrawal through VR, they had not experienced this themselves.

Eight participants, particularly participants using VR intervention II, indicated that VR provided relaxation. As participant V057 (51 yr, female, VR intervention group II) noted: “*I really enjoyed it. It was very relaxing, and I especially liked the meditation to relax*”**. This effect occurred immediately after starting the intervention, as mentioned by participant V022 (52, female, VR intervention group III): “*It’s a bit of a highway inwards. It’s nice to relax in a super-fast way*”**. Two participants appreciated that VR made them fall asleep.

Seven participants appreciated being able to apply VR to escape from the (negative impulses of the) surroundings or other patients in a shared room. This was reported most by participants in VR intervention groups II and III and reported least by participants in VR intervention group I. As participant V042 (57 yr, female, VR intervention group II) described: “*I found that very pleasant, especially in a 4-person room. You kind of have the feeling of having a bit of privacy*”**. Two participants disliked complete closure since it prevented them from engaging with their surroundings.

Three participants in VR intervention group III appreciated the immersive nature of VR, which created a feeling of presence in the virtual environment. Only one participant from VR intervention group II and none from VR intervention group I mentioned this. As participant V023 (67 yr, female, VR intervention group III) mentioned, “*I was sitting in that chair, and I really felt losing my slippers into the water, the experience is so real*”**.

Side effects were mentioned as a negative effect of VR. Greater immersion in the virtual world seemed to be associated with more side effects. No side effects were mentioned by participants in VR intervention group I. In VR intervention group II, two participants mentioned fatigue. In intervention group III, two participants again mentioned fatigue as a side effect, and two participants mentioned dizziness. The side effects were not a reason to stop using VR. Some participants switched applications after experiencing side effects.

Four participants reported that the effects of VR only lasted for a short period of time. Only one participant in VR intervention group II mentioned that he still felt relaxed for a long time after using VR. Two patients in intervention group III specifically reported that the transition from VR to the hospital environment was large. As participant V022 (52 yr, female, VR intervention group III) described: “*In fact, when I took off the glasses, it was like falling back into my body*”**.

### 3.3. VR Hardware and Software

The type of hardware markedly affected participants’ willingness to use VR. Items affecting the experience of participants included comfort, technique, and ease of use. We compared two types of hardware: the 2D hardware used in VR intervention group I and the 3D hardware used in VR intervention groups II/III (Table 1).

The hardware of VR intervention group I generally seemed to be comfortable. Only two participants indicated some discomfort with the headband. The weight of the headset and the headbands affected feelings of comfort, particularly for participants in VR intervention groups II/III. Three participants in these groups considered the headset too heavy, and five mentioned that the elastic headbands were unpleasant, being either too tight or too loose. Also, participants from these groups considered it difficult to wear the hardware in combination with glasses or a hairpiece.

Another disadvantage was that the glasses of the VR device became foggy, as mentioned by participant V041 (41 yr, male, VR intervention group III): “*It’s quite hot in the hospital, and because the goggles close completely, the glasses became foggy, requiring frequent cleaning and wiping during use*”**. Remarkably, this experience was shared only by participants from VR intervention group III, while the same hardware was used in intervention group II.

Major differences existed between the three interventions concerning the immersion level of the devices. VR intervention group I appreciated the immersion level the least because of its 2D view, contrasting to the 3D view of the device used in VR intervention groups II and III. The hardware of VR intervention I would benefit from a change in view (3D instead of 2D). As participant V070 (74 yr, male VR intervention group I) reported: “*It would be nice if you were really in the middle of it, instead of watching from a distance*”**. Also, the visual quality of this intervention was critiqued by two participants. The 3D view of VR intervention II was appreciated. However, not being able to move around in the 3D world was seen as a disadvantage compared to VR intervention III, by two participants.

The design of the hardware also affected the ease of use. Generally, all participants considered the devices easy to use and patients felt in control of VR. The device used in VR intervention group I was considered to work intuitively through the remote control buttons, while VR interventions II/III required a learning curve. Participants in VR intervention groups II/III referred to problems such as navigating through the menu or within the applications (eight times) and correctly using the controller (four times). Specifically, in VR intervention group II, three participants reported problems with switching navigation from the controller to navigating with the eyes or vice versa. Ease of use could be improved by a better connection between controller and headset, a more ergonomic controller for button usage, and fewer delays in navigation. 

Hardware could also be improved with a more powerful battery. The batteries of both the headset and controller of VR intervention groups II/III quickly emptied during use, and the need to recharge the batteries forced three participants to stop VR.

In all intervention groups, participants needed the paper manual while using VR. Two participants also needed personal support, especially as the manual may have been difficult to understand. As V050 (66 yr, female, VR intervention group III) described: “*I have read the instructions a number of times, but when you have had anesthesia you start to think very slowly and then it is difficult to understand it*”.

For VR software, interviews focused on patients’ preferences to understand what type of content and to what extent of the software catalogue should be offered after surgery. The type of content played seems to vary greatly per individual, independently of the randomized intervention. Preferences seemed to depend on various factors, including the level of immersion, the level of interaction, and the ease of engaging with an environment. Many participants would expand the catalogue with applications related to individual interests, such as their hobbies or job.

Participants in VR intervention group I found the catalogue limited, especially in terms of variety. Participants desired voiceovers to the nature videos, instead of classical background music, and suggested watching documentaries. In VR intervention group II, four participants indicated that the catalogue was fine. Participants suggested interactive applications that allow for “walking around” instead of exploring the environments from specific viewing points, as well as adding other types of content, such as nature documentaries. Two participants indicated that they would use VR more often if the catalogue was larger. As participant V042 (57 yr, female, VR intervention group II) stated: “*At that moment there were enough environments for me. But I only used it for 2 days. Perhaps if you had to use it for a longer period, it would be nice if you had more choice*”**. Most participants in VR intervention group III appreciated the size and variety of the catalogue, but they desired additional documentaries related to personal interests such as art, culture, nature, or space, as well as videos or environments representing their home environment.

Five participants remarked that adding content should not affect the ease of use of navigating between the various applications. Suggestions were made on adding a search function to the VR headset for better navigation and using an algorithm for predicting patient preferences and suggesting personalized content. As participant VS015 (52 yr, male, VR intervention group II) noted: “*Look, if you link something like a YouTube channel to it, you can offer many more applications that are focused on the person, for example, a pop concert or a nature program, something in the woods, etcetera*”.

### 3.4. VR Prescriptions

This theme included questions on the ideal number and duration per session, timing of VR sessions as well as the target population. 

In total, 12 out of 17 participants mentioned 2–3 sessions a day as the ideal number. In both VR intervention groups II and III, one participant desired to use VR 5–6 sessions a day considering normal care routines. Two-thirds of participants considered 10–15 min per session as an ideal length. Two participants preferred sessions up to 30 min, and in VR intervention groups I and III, one participant desired to use up to 60 min per session. In VR intervention group I, participants watched one or more applications per session, depending on their appreciation of the content. In VR intervention group II, only one participant viewed one application, while the other five switched applications. In VR intervention group III, three participants used one application, and four switched applications.

Participants preferred the use of VR at different moments during the day. However, due to the normal care routines in the hospital, the afternoon and evening were indicated as the most suitable. Two participants indicated using VR specifically at night to improve sleep, including V041 (41 yr, male, VR intervention group III): “*I like that you can easily put the device on at night. Because the night after surgery, I couldn’t sleep because of the pain*”**.

Participants also commented on the target population of VR. Five participants mentioned that patients should not be too ill to use VR. In line with this, three patients mentioned that the pain should not be too intense. Patients with a history of chronic pain or frequent use of analgesics reported that their medical history did not affect their willingness to use VR. Whereas most participants were above the age of 50, they considered children and adults under 50 to be ideal age categories for benefitting from VR. The reasons mentioned were that the distraction may work better for children and younger participants and their ability to use technology would generally be higher.

### 3.5. Implementation of VR

Participants were questioned on how they would ideally implement VR in hospitals, while sharing their experiences and ideas on the current healthcare context. 

Participants indicated that VR should always be within reach of the bed. As patients might be unable to leave their beds, they should be able to reach the VR themselves. In addition, two participants desired a wrist strap for the controller to prevent it from falling, as some patients could not reach the floor.

To ensure the optimal use of hardware and software, all participants appreciated the available support service delivered by the research team and would recommend continuing such a service in the future.

It was also recommended to dedicate time to using VR during the day, since the busy daily schedule of participants prevented them from using VR whenever they desired. The majority of participants indicated that their days were too full to be able to use VR for the advised number of three sessions, and eight participants appreciated scheduling VR sessions in their daily program. Three participants suggested the use of a ‘do not disturb’ sign while using VR since interference during a VR session was experienced as disturbing. As participant V042 (57 yr, female, VR intervention group II) noted: “*Someone was standing next to me, and he called my name, and then I heard it. But I had no idea how long I was wearing the device with him next to me. So yes, you are indeed shocked*”**.

The final recommendation of participants related to managing the expectations of patients and increasing the awareness of VR among patients and healthcare personnel. Participants reported that they wished to be informed on VR interventions prior to their hospital admission and surgery so they would know what to expect during recovery. Participant V041 (41 yr, male, VR intervention group III) indicated that the knowledge of using VR during admission would be something to look forward to: “*Oh wait I’m going to the hospital, and I can get virtual reality therapy to recover. Yes, that’s cool, I’d say a unique selling point*”**. Increasing the awareness of VR usage during hospitalization by healthcare personnel would reduce the refusal of use among patients, as V059 (53 yr, male, VR intervention group I) indicated: “*It is mainly the patients and perhaps also the staff who can be a problem. It should become much more widely known, then people will be less likely to say no, or people want to try it more often*”**.

Recommendations on the optimal use and implementation of VR, derived from patients’ experiences and preferences, are summarized in Table 3.

## 4. Discussion

### 4.1. Summary of Results

We investigated experiences and preferences for self-administered VR in pain management after major surgery. In order to optimize the clinical use of VR in accordance with patients’ needs and expectations, we interviewed 17 patients on their opinions on VR effects, hardware, and software, and recommendations on prescriptions and the implementation of VR. Most patients appreciated VR for its positive effects on pain, distraction, relaxation, privacy, and escape from surroundings, without experiencing serious adverse events. All participants would recommend VR to other patients, and the majority of patients would use VR again. The interview results contributed to a better understanding of the suitability of patients and VR interventions for VR pain treatment after major surgery, the deployment of VR at the surgical ward, and the support needed from hospital staff. 

### 4.2. VR Hardware and Software

Interestingly, we could not identify a certain type of application that was most suitable for patients. Suggested applications to expand the software catalogue were related to a wide variety of individual interests and themes. In particular, in the case of using VR for a longer period of time, it is therefore highly recommended to provide a large software catalogue that could meet the various needs and preferences of patients.

Another important consideration is the ease of using the HMD by postoperative patients. A considerable number of hardware issues were reported by participants in all intervention groups. Particularly, discomfort wearing the HMD and difficulties in navigating the VR applications were reported. The challenges of using VR by a patient feeling generally sick due to the operation should not be underestimated, and this requires the redesign of HMDs and applications in co-creation with patients. We previously reported comparable VR treatment challenges and described the recommendations of patients for adapting the VR hardware and software for self-administered VR at home [15,16]. More intuitive use of HMDs and applications increases the willingness and self-efficacy of patients to use VR, facilitates clinical implementation, and may reduce the workload of clinicians. In the past years, HMDs have developed into high-quality devices with fewer technical issues; however, practical shortcomings, particularly comfort levels and ease of use, also need to be addressed to facilitate implementation and increase the willingness of patients to use VR [17,18].

### 4.3. VR Prescription

An important novelty of this study is the use of VR as a self-administered approach. We allowed patients to use VR when needed, according to their own preferences, and on three consecutive days. This contrasts with previous studies that standardized VR usage by providing limited content in a single VR session or a limited number of sessions, supervised by a researcher [17,19,20]. The approach to only advise patients and let them freely choose VR usage allowed us to obtain insights into the patient-initiated use of VR, including the most suitable content and VR prescription from the patient perspective, which is a knowledge gap in clinical VR [5]. Although the prescription of 2–3 sessions per day with a session duration of 10–15 min was comparable to the advice according to the study protocol, it turned out that the preferred time of day for VR use differed between patients. Most patients preferred to use VR in the afternoon or evening hours, but some patients used VR mainly during night hours. This difference in preferences affects how HMDs are offered, which should be offered at the bedside and around the clock.

### 4.4. Implementation of VR

The patient experiences provided key considerations for the clinical implementation of VR. The first consideration is the introduction of VR to patients. Due to the study design, selecting patients with a postoperative pain score of 4 or higher, screening and inclusion had to be performed the day after surgery when patients felt generally sick, were focused on standard postoperative treatment, and were not prepared for non-pharmacological pain treatment with a VR device. Consequently, many patients refused to participate in the trial. Preoperative information and education on VR pain management could have changed patients’ expectations and increased preparedness for using VR more often, as reported in previous studies [20,21,22].

The second consideration is the workflow at the ward. Patients noticed the involvement of various clinicians and many activities aimed at recovery; however, to such an extent that only a few opportunities existed for an uninterrupted VR session. Due to interruptions, patients had to discontinue VR sessions, could not apply VR as frequently as they preferred, or had to postpone VR sessions to the evening or night hours. This barrier was mentioned by patients in both hospitals and exposes the uncoordinated workflows from a patient perspective, hampering self-efficacy in managing pain. Simultaneously, clinicians visiting the ward were not well informed about the study and were unaware of the potential of VR in pain treatment. The implementation of VR should therefore focus on both patients and healthcare personnel by raising awareness of VR in pain management and emphasizing the importance of ‘VR time’ for patients who can benefit from VR.

### 4.5. Limitations

This study has some limitations to be considered. First, it is important to note the sample of patients included in this substudy. This study was conducted in a specific patient population of postsurgical, hospitalized patients after major surgery, potentially limiting the generalizability of the findings to other healthcare contexts or patient populations. On the contrary, the large number of participating surgical departments has prevented the application of the results to a specific surgical setting. The average age of participants was 58, with an age range from 39 to 74, indicating a relatively older age group of participants. The experience and appreciation of this technology in these patients, who can be classified as ‘digital natives’ or ‘digital immigrants,’ can differ from digital native individuals, i.e., individuals who grew up with digital technology as part of their daily lives. This potentially limits the generalizability of the findings; however, it should be mentioned that the age range of patients included in the interviews represents today’s average surgical population. It should also be mentioned that the majority of interviews included patients from one hospital. Although additional interviews with patients in the second participating hospital did not reveal new themes or topics, this may also limit the generalizability of the findings to other hospitals.

Second, this study was conducted as a substudy of a randomized controlled trial, and the design and complexity of the main trial may have discouraged patients from participating in the trial and substudy. In addition, participants who agreed to be interviewed might have had positive experiences with VR, potentially biasing the results toward more favorable perceptions. It should be mentioned, however, that patients were also interviewed on negative experiences or negative feedback concerning the interventions, and that all patients randomized for a VR intervention (starting from 20 randomizations), were asked to participate in the interviews, thereby minimizing bias as much as possible.

The current study focused on the experiences of patients, whereas clinicians and other staff were not involved in the interviews. Individual caregivers had little exposure to patients who used VR for meaningful contribution in this study. However, they may have had valuable input on the integration of VR in regular postoperative care, as well as the service and support activities, including technical assistance. Patients emphasized the importance of such a service for the routine clinical use of VR. Recommendations on VR implementation from the patient’s perspective also lack information on the practical aspects of VR interventions, including the distribution, collection, and cleaning of VR devices. It is recommended to consider these organizational aspects of VR, but the ideal operating procedure and governance need to be determined. Also, experiences were collected per patient and from five individual patients per HMD and the accompanying applications. Focus group interviews might have revealed more topics and more detailed information on shared patient opinions. However, this is difficult to organize during hospital admission and with different parties involved [23].

### 4.6. Future Perspectives

The results of this study can contribute to the implementation of VR postoperative pain self-management. However, questions remain unanswered regarding the seamless integration of VR in postoperative care and workflow, the appropriate service system, the effects on the workload of staff, and, ultimately, the health-economic aspects. Based on a 30 min reduction of nurse time per day for managing pain in surgical patients, we calculated a financial benefit within 3 years of using a hospital-wide VR service unit (unpublished data). Previously, the length of stay, patients’ satisfaction, and opioid reduction were considered as outcomes in a cost-effectiveness decision model analysis of implementing VR as an adjuvant pain therapy among hospitalized patients in general, with a reduction in the length of stay as the primary cost-saving outcome [24]. We recommend considering multiple outcomes in the patient, caregiver, organizational, and societal domains for the further health economic assessment of VR postoperative pain management, for which the results of the present study can be useful.

## 5. Conclusions

We conclude that hospitalized surgical patients could benefit from pain self-management using VR. However, we identified different challenges in fulfilling individual needs, embedding the treatment into the routine clinical workflow, and (technical) support. Patient experiences can help to reveal important aspects of a successful clinical implementation of VR as a non-pharmacologic means of pain treatment. Recommendations from this study can be used for future VR use and research.

## Figures and Tables

**Table 1:** Overview of the Virtual Reality (VR) intervention subgroups.

Intervention Group	I: Passive 2D VR Intervention	II: Intermediate 3D VR Intervention	III: Interactive 3D Intervention
	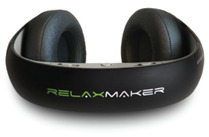	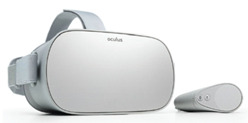	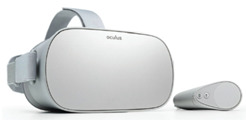
Hardware	Relaxmaker ^1^ 2D 120° view	Oculus Go ^2^ 3D 360° view	Oculus Go ^2^ 3D 360° view
Software Number and duration * of applications Type of applications * Interventions could be stopped anytime	10 videos; 20–55 min Relaxmaker 2D nature videos with landscapes and animals, natural sounds combined with classical background music.	10 environments; ≥5 min CareVRx ^3^: 360° nature video shots and guided imagery experiences, natural sounds combined with classical background music.	19 applications; ≥5 min Oculus Go Consumer applications, freely available: Nature videos, virtual nature experiences, guided meditation, games for racing or shooting, applications for exploring tourism hotspots, museums, or languages.
Immersive	No	Yes	Yes
Interactive	No	Intermediate; participants could explore 360° virtual environments by changing the point of view in the environment.	Yes, level of interaction varied between applications including 3D 360° videos, exploring a 360° virtual environment, and playing interactive 3D VR games.

^1^ RelaxMaker, Beter door beeld, Terwolde, The Netherlands. ^2^ Oculus Go, Facebook Technologies, Irvine, CA, USA. ^3^ C.A.R.E.^®^ VRx, Healing Healthcare Systems, Reno, NV, USA.

**Table 2:** Characteristics of the interviewed patients.

Interview	VR Group	Gender	Age	Level of Education	History of Chronic Pain *	Type of Surgery	Number of Days Using VR	Number of VR Sessions	Total Time in VR (min)
1—V022	III	Female	52	Bachelor’s degree	Yes	Abdominal surgery (laparotomy)	3	11	255
2—V023	III	Female	67	Bachelor’s degree	Yes	Abdominal surgery (laparotomy)	3	5	70
3—V029	II	Male	72	Bachelor’s degree	No	Abdominal surgery (laparotomy)	3	7	95
4—V030	I	Female	62	Vocational education	Yes	Trauma surgery (lower extremity)	3	7	175
5 —V033	III	Male	71	Bachelor’s degree	Yes	Trauma surgery (lower extremity)	3	4	95
6—V038	I	Male	54	Vocational education	No	Orthopedic back surgery	3	3	35
7—V041	III	Male	41	University education	Yes	Orthopedic hip surgery	3	10	315
8—V042	II	Female	57	Bachelor’s degree	Yes	Trauma surgery (lower extremity)	2	3	55
9—V043	II	Male	39	Vocational education	No	Trauma surgery (lower extremity)	3	5	60
10—V044	II	Female	62	Vocational education	Yes	Orthopedic knee surgery	3	5	44
11—V050	III	Female	66	Vocational education	Yes	Orthopedic hip surgery	3	10	144
12—V057	II	Female	51	University education	Yes	Trauma surgery (lower extremity)	3	8	225
13—V059	I	Male	53	Bachelor’s degree	Yes	Abdominal surgery (laparotomy)	1	1	10
14—V066	I	Male	57	Vocational education	Yes	Trauma surgery (lower extremity)	2	8	100
15—V070	I	Male	74	Bachelor’s degree	No	Abdominal surgery (laparotomy)	3	10	132
16—VS012	III	Female	55	Vocational education	Yes	Orthopedic knee surgery	3	11	235
17—VS015	II	Male	52	Vocational education	Yes	Orthopedic back surgery	2	3	40

VR: Virtual Reality. * Chronic pain: any pain lasting for over three months, pain not necessarily related to the indication for surgery.

**Table 3:** Summary of recommendations on Virtual Reality (VR) in pain management divided into three topics: VR hardware and software, VR prescription, and VR implementation.

VR hardware and software	A 3D, 360-degree device, including full coverage of view is preferred, but in some cases, such as nausea or anxiety, non-immersive VR could be more suitable. VR devices should include intuitive user interfaces, excellent Wi-Fi connections, and long-lasting batteries. VR devices must be comfortable to wear. The optimal software catalogue contains multiple immersive and interactive applications, from which patients can select applications based on their personal interests. The software catalogue should be suitable for long-term use, if indicated.
VR prescription	Optimal dosage and frequency are 2–3 sessions of 10–15 min a day. Provide the possibility for individual adjustment of VR prescriptions. Give patients control over when and how to use VR. VR is less suitable for patients who experience severe pain, nausea, or fatigue. A history of chronic pain and/or analgesic use does not hinder the use of VR.
VR implementation	Provide VR time for patients to avoid interruptions during VR use. Ensure the VR headset is stored within reach of the patient. Provide a manual and support service for assistance (remotely). Inform patients in advance about the possibility of using VR. Involve healthcare personnel in implementing and using VR.

## Data Availability

The datasets used and/or analyzed during the current study are available from the corresponding author on reasonable request.

## Supplementary Materials

The following supporting information can be downloaded at: https://www.mdpi.com/article/10.3390/jcm12216805/s1, Supplementary Materials: interview questionnaire, including S1: topic list and S2: interview themes.
