# Supplementary material for: Self-Administered Virtual Reality for Postsurgical Pain Management: A Qualitative Study of Hospital Patients’ Reported Experiences
Source: J Clin Med. 2023 Oct 27;12(21):6805. doi: 10.3390/jcm12216805 (PMC10647437; doi:10.3390/jcm12216805)
Supplement: Supplementary file 1 [file jcm-12-06805-s001.zip › jcm-2641305-supplementary.pdf]

Supplementary Materials: Interview questionnaire

S1. Topic list

a. VR in general
b. Experiences of VR related to effects
c. Software
d. Hardware
e. VR prescriptions
f. Implementation of VR

Interview questionnaire (related to topic)

1. How did you experience VR? (a,b)

2. What type of application did you enjoy the most? Could you tell why? (c)

3. What type of application did you like least? Could you tell why? (c)

4. Did you find the available catalogue of Virtual Reality applications large enough? (c)

5. If you could choose, what type of applications would you like to add to the catalogue? (c, f)

6. What is the ideal number of times a day for you to use Virtual Reality? (e)

7. For how many minutes would you prefer to use Virtual Reality at a time? (e)

8. Do you prefer multiple applications each time you use Virtual Reality, or just one application at a time? Could you tell why? (e)

9. Did you encounter any problems while using Virtual Reality? What problems were these? What would have to change to solve these problems? (d,f)

10. Have you received enough help to be able to use Virtual Reality? What did you need help with? Were there times when you would have liked more help? (f)

11. Was the instruction form clear? If not, what could be modified to improve the instruction form for unsupervised use of Virtual Reality? (f)

12. How did you experience the Virtual Reality device while wearing them on the head? (d)

13. Did you feel you had sufficient control over the use of the Virtual Reality? If not, what was the cause? (d)

14. Do you feel familiar with the Virtual Reality experiences? (b,c)

15. What was the effect of Virtual Reality on the pain you experienced? What type of application did you find most effective for pain relief and why? Which type of application did you find to be the least effective for pain relief and why? (b)

16. What is your opinion on pain relief through Virtual Reality? Are there any advantages or disadvantages? (b)

17. Did your opinion on pain relief through Virtual Reality change during the study? If so, how? (a)

18. Has Virtual Reality also had other effects? (b)

19. Do you have an idea how we can improve the use of Virtual Reality by patients? (f)

20. If you find yourself in the same situation, would you like to use Virtual Reality again in the future? (a)

21. Would you recommend Virtual Reality to others? (a)

22. Do you have any questions or comments? (a)

S2. Interview themes

**Main topics**	**Themes* and subthemes-**
Experiences of VR related to effects	* VR in general:<br>- General opinion<br>- Use of VR in the future<br>- Recommend VR to others<br>* Pain reduction<br>* Use of analgesic medications<br>* Relaxation<br>* Distraction<br>* Immersion<br>* Sleep<br>* Isolation and privacy<br>* Adverse effects<br>* Duration of effects<br>* Comparison to other audiovisual devices<br>* Effects per application or environment
VR Software	* Positive opinions on catalogue of applications and environments<br>* Negative comments on catalogue of applications and environments<br>* The number of applications:<br>- sufficient<br>- too small<br>- too large<br>* Recommendations on software catalogue<br>* Personal profiles and application preferences
VR Hardware	* Fit:<br>- Comfort<br>- Weight<br>- Elastic strap belt<br>- Warmth<br>- Combining device with glasses or hairpiece.<br>* Technical features<br>- View 2D / 3D<br>- Quality of the view<br>- Quality of sound<br>- Battery<br>- Errors<br>* Usability<br>* Ease to use<br>* Problems during use<br>* Control over VR<br>* Instructions and manual<br>* Recommendations on hardware

VR prescriptions	* Frequency<br>* Duration<br>* Number of applications per session<br>* Timing<br>* Target population
Implementation of VR	* Device location in patient’s room<br>* Support service<br>* Daily time schedules<br>* Implementing VR in normal care routines<br>* Awareness among patients and clinicians
